# Attitudes Towards Food Allergy Scale: Psychometric properties and associations with food allergy quality of life and anxiety

**DOI:** 10.1002/clt2.12205

**Published:** 2022-10-25

**Authors:** Gabriel Lins de Holanda Coelho, Renan Pereira Monteiro, Audrey DunnGalvin

**Affiliations:** ^1^ Postdoctoral Researcher at University College Cork Cork Ireland; ^2^ Federal University of Paraíba Joao Pessoa Brazil; ^3^ School of Applied Psychology University College Cork Cork Ireland

**Keywords:** attitudes, food allergy, measure development, psychometrics, quality of life

## Abstract

**Background:**

Daily, we tend to evaluate things positively or negatively, according to whether they follow the general information available about them. This attitudinal assessment is represented through evaluative dimensions (e.g., good‐bad) that vary in terms of valence (positive or negative) and strength (less or more). Despite its importance, there is an urge in food allergy (FA) research to properly assess attitudes based on the underlying mechanisms that define attitudes.

**Objective:**

The present research aimed to develop the Attitudes Towards Food Allergy scale (ATFAS), the first attitudinal measure of FA. Method: Two studies were performed (*n* = 1049), using a range of robust statistical analyses (e.g., Item Response Theory, Exploratory Factor Analysis, Confirmatory Factor Analysis).

**Results:**

Our results provided strong evidence for a unidimensional attitudinal structure, across groups of non‐allergic individuals and food‐allergic, besides recommended reliability levels. All items presented suitable parameters (i.e., discrimination, difficulty, information). Finally, the ATFAS significantly predicted FA quality of life, mediated by FA anxiety.

**Conclusion:**

We are confident that the ATFAS is a novel and necessary measure, that can help to widen how we view and assess FA. The development of studies that assess attitudes towards FA based on our general information about the disorder would help to deepen our understanding of their links to other health‐related variables and their potential impact on quality of life, reduce FA's stigma, and develop more positive attitudes.

## 1 INTRODUCTION

Food allergy (FA) is responsible for 90% of allergic reactions worldwide, making it a significant public health concern with high costs to the public service.^1^ The impact of FA goes beyond the disorder itself. It raises a concern about what needs to be considered to improve food allergy quality of life (FAQL), since it is likely that the prevalence of FA will continue to increase in the short to medium term.^2^ The importance of addressing such concerns and improving the FAQL of those directly and indirectly affected by the disorder is evident when assessing its associations to different psychological parameters/components. For instance, FAQL is linked to general anxiety in children and their mothers,^3^ and FA‐specific anxiety in adults.^4^ Also, parents perceive a lower impact of FA on the FAQL of their children when compared to the children's views.^5^ Finally, parents of children with FA presented increased levels of anxiety, depression, and stress compared to parents of children with no allergies.^6^ Therefore, it is necessary to investigate how people with FA and those indirectly impacted by the disorder, such as parents and caregivers, cope with their burden, the psychological variables that can help diminish the FA impact, and how we can improve their well‐being. One of these variables is attitudes, yet to be studied more in‐depth in FA research. The present research aimed to develop the Attitudes Towards Food Allergy scale (ATFAS), the first attitudinal measure of FA.

### 1.1 Attitudes

Attitudes can be defined by the psychological predisposition to assess something favourably or unfavourably.^7^ For instance, if individuals are motivated to try new things, they will have positive attitudes towards experimenting with food that is not typical in their culture or country. From a psychological perspective, an individual develops this ability based on a combination of cognitive, affective, and behavioral knowledge.^8^ This attitudinal assessment is represented through evaluative dimensions (e.g., good‐bad) that vary in terms of valence (positive or negative) and strength (less or more).^9^ The influence of attitudes has been widely assessed. For example, individuals who endorse excitement values are more likely to have a more positive attitude towards the use of drugs.^10^ Moreover, individuals with stronger attitudes to environmental preservation present a higher future‐focused time perspective (that is, they care more about the planet's future).^11^ Finally, a meta‐analysis shows men have more positive attitudes toward technology than women.^12^

These examples highlight the multidisciplinarity of attitudes within research. In health psychology, research has shown that attitudes can be indicators of both behaviors and psychological outcomes. For example, individuals with more severe types of epilepsy have more negative attitudes towards the disorder, with these negative attitudes being linked to most severe depression and lower self‐esteem.^13^ Moreover, certain attitude types have been associated with particular coping strategies for respiratory illness.^14^ Furthermore, more positive or negative attitudes towards individuals on the autistic spectrum were related to a higher or lower intention for social interaction.^15^

Despite the significant findings in different fields and across various disorders, the exact impact of an attitudinal assessment of FA is unknown. To date, research on attitudes in FA has tended to conflate attitudes with behavioral response, beliefs, or ‘opinions’, variables that are themselves influenced by attitudes.^16^, ^17^ These researchers have focused on how participants react to a specific event or their beliefs on what should be done if something happens. For instance, in a study that aims to assess parental knowledge, attitudes, and beliefs regarding FA, the topics are blended to refer to knowledge or opinion, such as on whether children should carry an EpiPen or whether the school should have trained staff.^16^ In another study, researchers aimed to assess the knowledge and attitudes about FA and anaphylaxis in a sample of primary care physicians.^17^ However, the topic is mispresented again, referring to general knowledge about the FA than proper attitudinal judgment. Despite these misinterpretations, it is vital to acknowledge the value of the extant literature. The psychological definition of attitudes may not be widely spread across different areas, so its use can be reduced to standard definitions.

### 1.2 The present research

There is an urge to properly assess attitudes based on the underlying mechanisms that define the topic. The development of studies that assess attitudes towards FA based on our general information about the disorder would help to deepen our understanding of their links to other health‐related variables and their potential impact on quality of life. Knowing these associations might lead to developing interventional strategies that aim to reduce the stigma that involves FA, and reduce its burden on those directly or indirectly affected. For instance, in interviews conducted with children, youth, and their parents, researchers found that the kids with FA felt stigmatized due to school policies, which impacted their physical safety and social well‐being.^18^ Therefore, helping to provide more positive attitudes towards a disorder might help reduce situations like this and improve daily quality of life.

The first step for a valid and reliable assessment is developing a psychometrical tool, as precise as feasible, to specifically assess attitudes toward the FA. Therefore, the present research aimed to develop the Attitudes Towards Food Allergy Scale (ATFAS), the first attitudinal measure of FA. For that, we performed two studies (*n* = 1049), using gold‐standard psychometrical procedures. Study 1 used a mixed sample of allergic and non‐allergic to food individuals. The inclusion of different samples was deemed necessary to assess whether the measure structure is replicable across groups and beyond those directly and indirectly impacted by FA. This might help, for instance, to understand the different groups' perspectives (such as food business owners and restaurant managers), toward the FA. In Study 2, we counted exclusively individuals with some FA. We used gold‐standard statistical techniques to assess the psychometric parameters of the measure (i.e., Exploratory Factor Analysis (EFA), Item Response Theory, Confirmatory Factor Analysis, reliability analysis). Moreover, we assessed its convergent validity by associating the ATFAS with FA anxiety and FAQL. Anxiety is known for having a significant impact on those living with FA,^3^, ^4^ and FAQL is an important research topic to help understand how the disorder impacts the well‐being of those, directly and indirectly, involved.^3^, ^5^, ^6^ Finally, this research is part of the FA Coping and Emotions (FACES) project, which aims to provide a more in‐depth assessment of the underlying mechanisms of living with FA (i.e., coping, psychological responses).

## 2 STUDY 1: METHOD

### 2.1 Participants and procedure

This study used a mixed sample of allergic and non‐allergic to food individuals. We recruited participants through the academic crowdsourcing platform Prolific (https://www.prolific.co/). To be included in this study, participants had to meet several pre‐screening requirements; birth country (the United Kingdom or Ireland), a minimum number of other surveys answered on Prolific (minimum of 25), and an approval rate of at least 98%. A second link was created, adding a pre‐screening requirement of being diagnosed with a FA to help differentiate the groups. A total of 630 individuals (*M*_age_ = 41.85; SD_age_ = 11.02; 444 women, 186 men) participated in the study, with the majority from the United Kingdom (*n* = 626). Of those participants, 418 did not have a FA, whereas 212 were diagnosed as allergic to at least one food allergen. Within the group with FA, peanuts (*n* = 54), tree nuts (*n* = 48), and shellfish (*n* = 39) were the primary allergens.

### 2.2 Material

Participants completed the ATFAS. The measure comprises six bipolar items, with opposing adjectives at each end (also known as semantic differential scale). The first four bipolar items (i.e., *bad‐good, unsatisfactory‐satisfactory, unfavorable‐favorable, negative‐positive*) were adapted based on the well‐known work from Armitage et al.^19^ We further included the other two items (i.e., *difficult‐easy, sad‐happy*), that we considered relevant in the FA context. Participants answer how they see FA using a bipolar seven‐point scale, from −3 to +3. Answers close to either end of the scale denote a more positive or negative response, whereas answers in the middle (zero) are neutral. The full scale is available as Appendix.

### 2.3 Data analysis

We performed the analyses using SPSS and the open‐source software *R*.^20^ In SPSS, we performed the EFA. To determine the number of factors, we considered the parallel analysis criteria. After, we performed the EFA, using the Principal Axis Factoring method. We examined the EFA results for each subsample (No FA and FA). After, using *R*, we assessed the individual parameters (i.e., discrimination, difficulty, information) of each of the ATFAS items using the *Multidimensional Item Response Theory* package.^21^ We used the graded response model^22^ to assess item parameters, in the case that ATFAS has an answer scale with more than two categories. Finally, we assessed the reliability levels of the measure through McDonald's omega (ω) and Cronbach's alpha (α), with recommended values over 0.70.^23^

## 3 RESULTS

### 3.1 Exploratory factor analysis and reliability

First, we assessed our data's suitability to perform the EFA, with results supporting its use (Non allergic, KMO = 0.90, Bartlett's Test (15) = 1414.37, *p* < 0.001; Food Allergic, KMO = 0.88 Bartlett's Test (15) = 648.87, *p* < 0.001; Overall, KMO = 0.90, Bartlett's Test (15) = 2016.14*, p* < 0.001). Moreover, the parallel analysis indicated the preference for a one‐dimensional structure. Therefore, we performed three EFAs for each subsample separately and together, with a one‐dimensional structure fixed. Table 1 shows that all items had a factorial loading over |0.65|, with the unidimensional factors explaining variance of at least 57.24%. Moreover, McDonald's omega and Cronbach's alpha levels were over the minimum recommended by the literature,^23^ indicating a reliable measure across the three groups.

**TABLE 1:** Exploratory factor analysis

Item	Non‐allergic (418)	Food allergic (212)	Full sample (630)
Bad—Good	0.828	0.843	0.834
Unsatisfactory—Satisfactory	0.706	0.693	0.703
Unfavourable—Favourable	0.790	0.805	0.793
Negative—Positive	0.806	0.845	0.818
Difficult—Easy	0.769	0.654	0.720
Sad—Happy	0.769	0.674	0.735
Eigenvalues	3.64	3.43	3.45
Explained variance	60.67%	57.24%	59.10%
Cronbach's alpha (α)	0.90	0.88	0.89
McDonald's omega (ω)	0.90	0.88	0.89
Average interitem correlation	0.60	0.58	0.59

### 3.2 Item Response Theory

Next, we assessed the individual item parameters of each item of the ATFAS: their level of discrimination, difficulty, and information. The discrimination parameter indicates to what extent an item can differentiate individuals with various latent trait levels^24^—in this case, attitudes towards FA. Therefore, a good item can distinguish those with negative, neutral, and positive attitudes towards the disorder. To assess the discrimination levels, we followed Baker's^25^ classifications. As shown in Table 2, all items showed very high discrimination levels (*a* > 1.7).

**TABLE 2:** Item parameters of the attitudes towards food allergy scale

Item	*a*	*b*1	*b*2	*b*3	*b*4	*b*5	*b*6	*b*(*m*)
Bad—Good	3.595	−0.618	0.19	0.659	1.789	2.188	2.79	1.17
Unsatisfactory—Satisfactory	2.388	−0.891	0.004	0.519	2.044	2.427	3.102	1.2
Unfavourable—Favourable	2.917	−0.456	0.324	0.923	1.732	2.138	2.739	1.23
Negative—Positive	3.328	−0.614	0.186	0.787	1.794	2.158	2.824	1.19
Difficult—Easy	2.174	−0.616	0.467	1.095	1.565	2.107	3.029	1.27
Sad—Happy	2.285	−0.913	−0.02	0.618	1.886	2.529	3.223	1.22

*Note*: *a* = discrimination; *b*1—*b*6 = threshold; *b*(*m*) = means between *b*1—*b6*.

Moreover, we assessed items' difficulty levels. Specifically, if an item is ‘too easy’, most participants will highly endorse it. In contrast, if an item is ‘too difficult’, only those with higher levels in the latent trait will fully endorse it. In this case, if an item is too easy, most participants will answer towards the positive end of the scale. In contrast, participants will answer towards the opposing end if an item is too difficult. Therefore, it is recommended that an item presents mean difficulty levels within the recommended threshold (−1.5 and 1.5), meaning they are neither too difficult nor too easy.^26^ As shown in the last column of Table 2, all items presented average difficulty levels within the recommended threshold.

**FIGURE 1:**
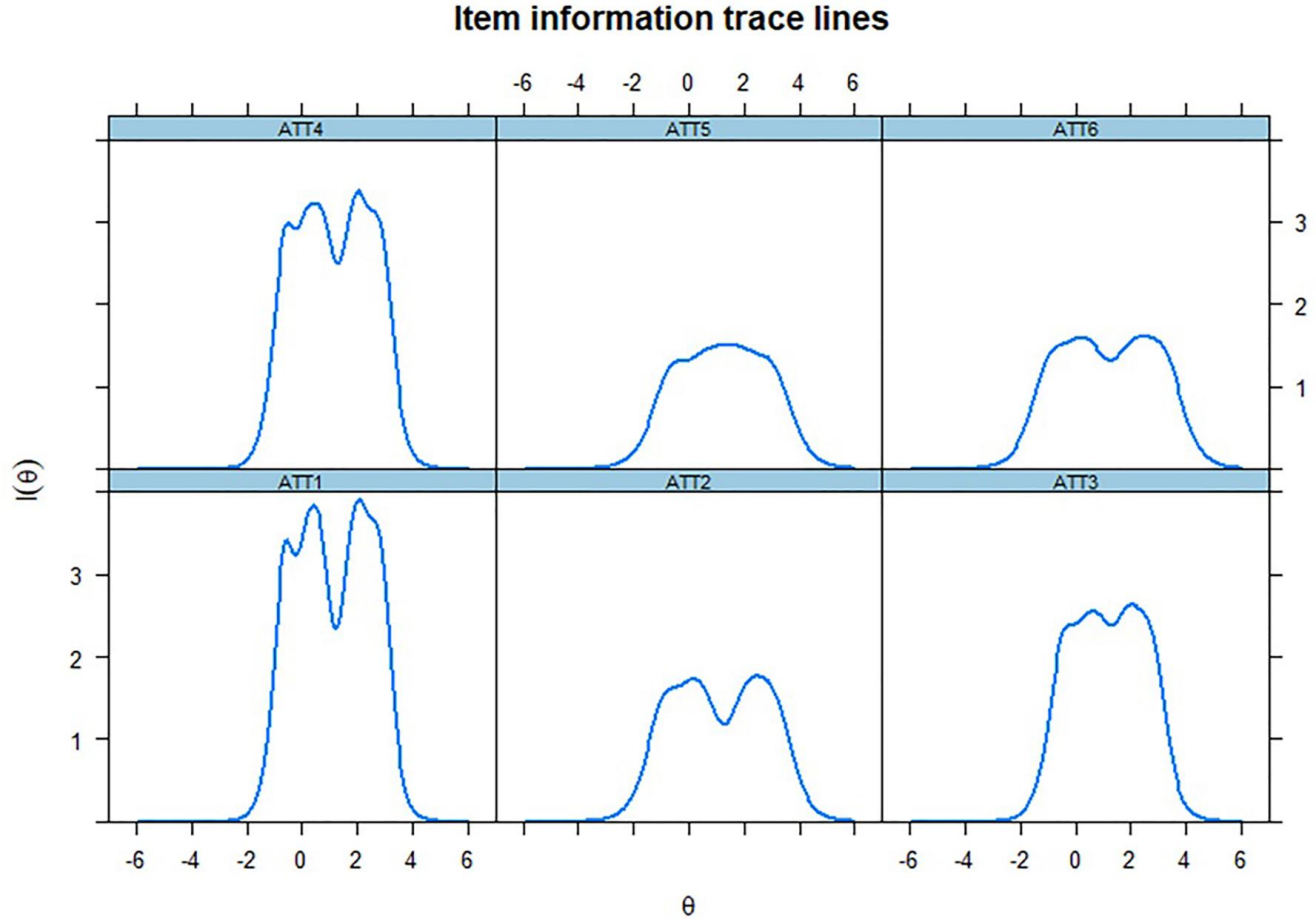
Item information curves of the Attitudes Towards Food Allergy scale (ATFAS)

**FIGURE 2:**
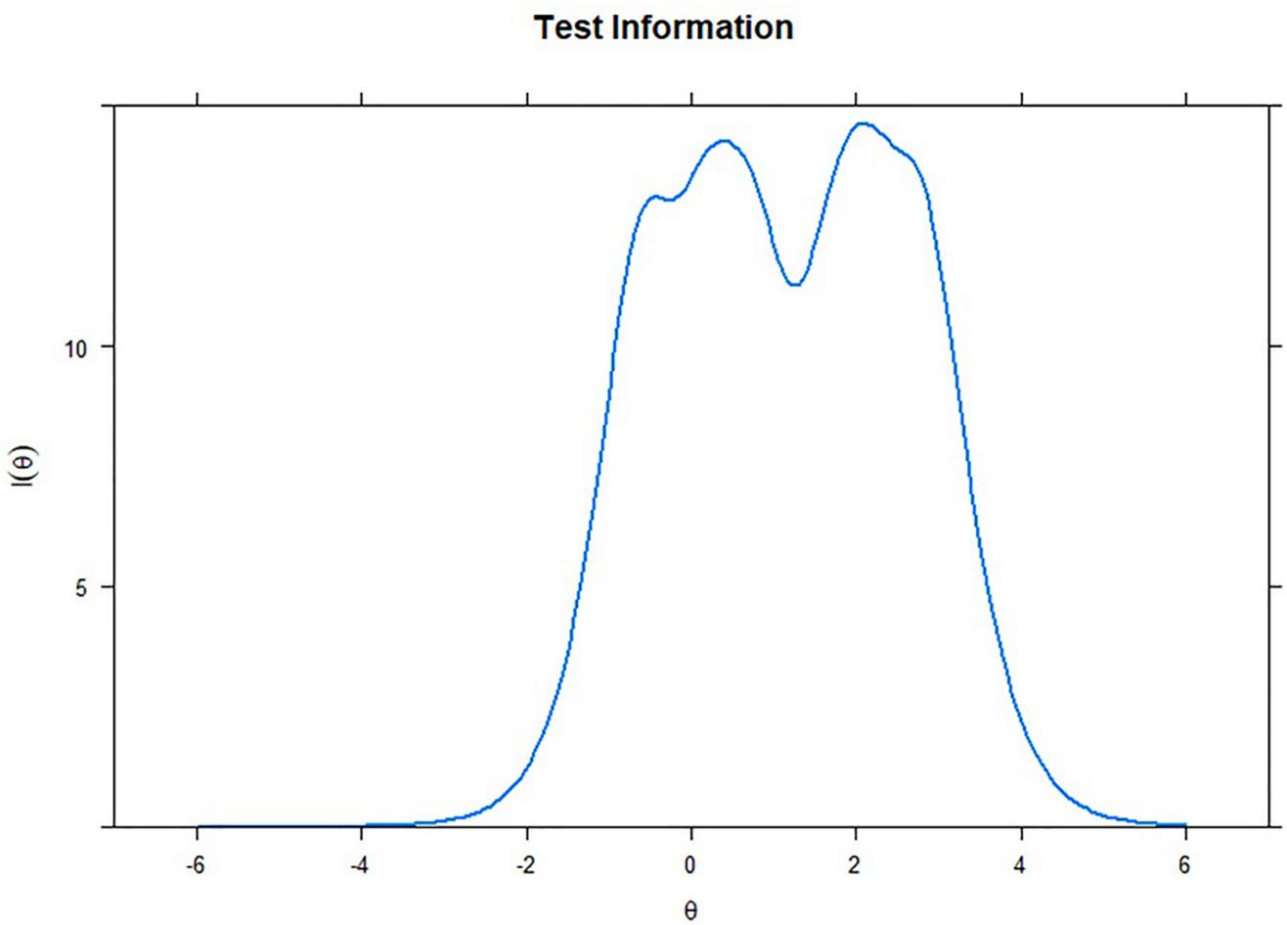
Test information curve of the Attitudes Towards Food Allergy scale (ATFAS)

Finally, we assessed each item's individual and joint contribution to the ATFAS using Item Information Curves (Figure 1) and Test Information Curve (Figure 2). More informative items contribute to a more informative measure, and therefore help to increase its reliability levels. For instance, the total information of 10 is similar to a reliability level of 0.90.^27^ Figures 1 and 2 show that all items contributed a reasonable amount of information, individually and together.

## 4 STUDY 2: METHOD

### 4.1 Participants and procedure

Participants were 419 individuals (*M*_age_ = 36.86; SD_age_ = 12.67; 299 women, 120 men) collected through Prolific. Besides the pre‐screening features used in Study 1, we also required participants to have a type of FA to be eligible to answer the survey. Participants who were included in the Study 1 were not allowed to participate. Most of these participants were from the United Kingdom (*n* = 397). Peanuts (*n* = 128), tree nuts (*n* = 112), and shellfish (*n* = 75) were among the most mentioned allergens. Finally, most participants were diagnosed by a general practitioner\family doctor (*n* = 149).

### 4.2 Material

Besides the ATFAS, we asked a subsample (*n* = 207; *M*_age_ = 36.83, SD_age_ = 13.17; 142 women, 65 men) to complete two additional questionnaires, to allow for assessment of convergent validity. First, the *Food Allergy Anxiety Scale*,^4^ the first FA‐specific questionnaire to develop the unique impact of FA on anxiety, with strong and suitable psychometric properties. The questionnaire is composed of 15 items (e.g., *I often think about having a reaction to food; I often feel that something bad will happen to me because of FA*), and participants have to indicate to what extent they agree to each of the items, using a five‐point scale (1 = *Strongly Disagree*; 5 = *Strongly Agree*).

Also, participants answered the *FA Quality of Life Questionnaire—Adult Form*.^28^ FAQLQ is the most widely used health‐related quality of life questionnaire in FA.^29^ The questionnaire is composed of 29 items, and participants indicate how troublesome\worried\frightened they are because of FA in various situations, using a seven‐point scale (1 = *Not*; 7 = *Extremely*). The FAQLQ covers four subscales: emotional impact (e.g., *How frightened are you because of your FA of an allergic reaction?*), FA health (e.g., *How worried are you because of your FA that the allergic reactions to foods will become increasingly severe?*), risk (e.g., *How troublesome do you find it, because of your FA, that you sometimes frustrate people when they are making an effort to accommodate your FA*?), social dietary limitations (e.g., *How troublesome do you find it, because of your FA, that you are able to eat fewer products?*). The FAQLQ results were reversed in our analyses so that higher values could indicate better FAQL.

### 4.3 Data analysis

All the analyses were performed on the free, open‐source software JASP (https://jasp‐stats.org/). We performed Confirmatory Factor Analysis using the Robust Maximum Likelihood (MLR) estimator. To assess model fit, we used the following indicators^30^, ^31^: Comparative Fit Index (CFI) and Tucker‐Lewis Index (TLI), with recommended values over 0.90; and Root mean square error approximation (RMSEA), with recommended values below 0.08. Once again, we assessed reliability levels using McDonald's omega and Cronbach's alpha. Finally, to assess convergent validity, we performed Pearson's correlation and developed a mediational model, using the Maximum Likelihood estimator and 5000 bootstrap simulations.

## 5 RESULTS

### 5.1 Confirmatory factor analysis and reliability

First, to assess whether the one‐dimensional structure from Study 1 holds, we performed a Confirmatory Factor Analysis. Results indicate a good model fit for the ATFAS: CFI = 0.98, TLI = 0.98, RMSEA = 0.058 (90% 0.033 −0.084). All the factorial loadings were statistically different from zero (λ ≠ 0; *z* > 1.96, *p* < 0.05), and ranged from 0.66 (*Difficult—Easy*) to 0.86 (*Negative—Positive*). Moreover, both McDonald's omega and Cronbach's alpha indicated a reliability level of 0.90, over the minimum recommended.^23^

### 5.2 Convergent validity

Using a subsample of 207 participants, we found significant relations between attitudes towards FA, FA anxiety, and the FAQL subscales. With FA anxiety, the correlation was negative (*r* = −0.38, *p* < 0.001), whereas for the FAQLQ subscales, all results were positive: emotional impact (*r* = 0.38, *p* < 0.001), FA health (*r* = 0.34, *p* < 0.001), risk (*r* = 0.32*, p* < 0.001), and social dietary limitations (*r* = 0.36, *p* < 0.001). That is, more positive attitudes towards FA is linked to lower levels of anxiety, and a higher FAQL.

**FIGURE 3:**
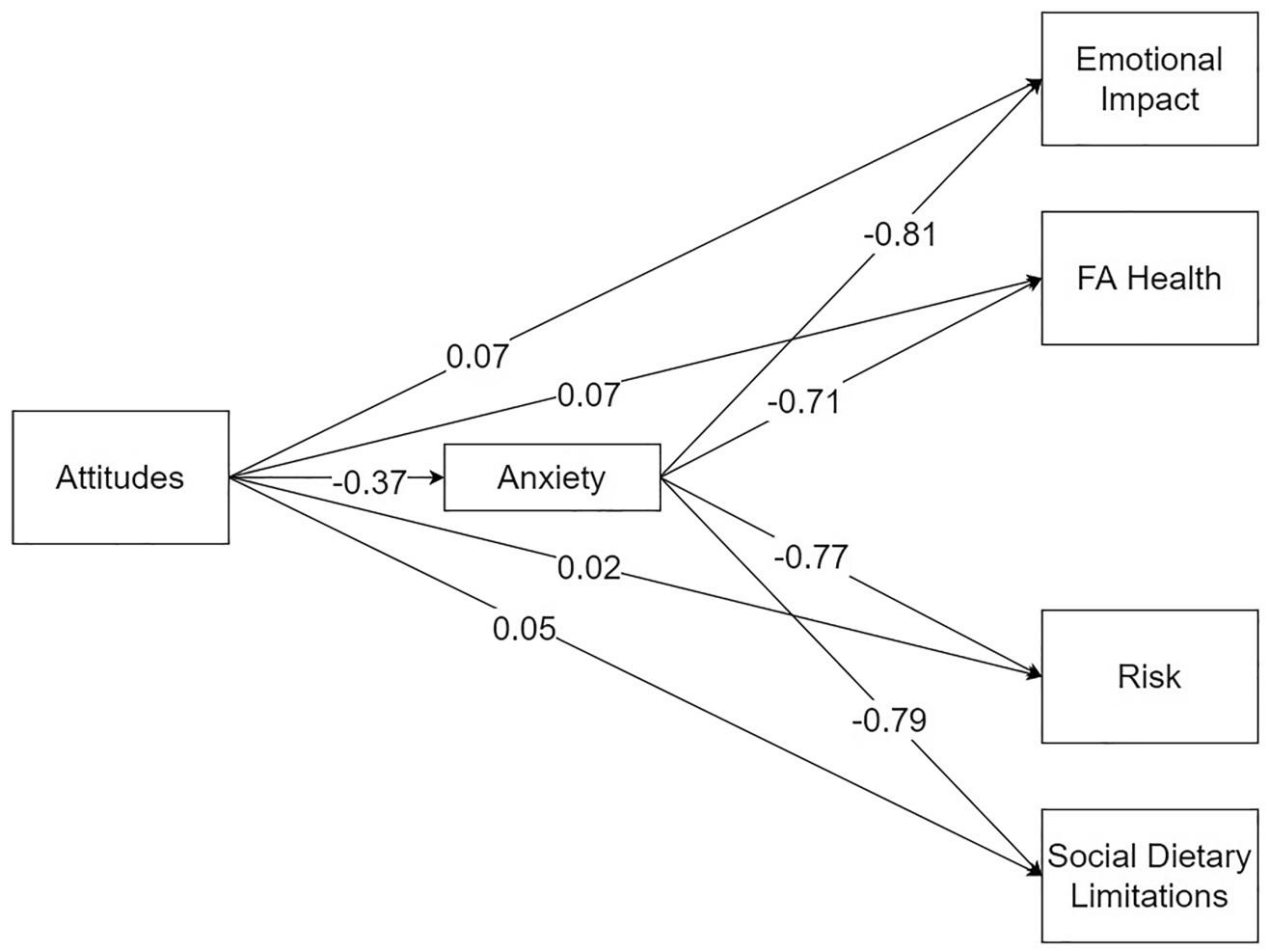
Mediational model (Attitudes Towards Food Allergy Scale (ATFAS), X → food allergy (FA) anxiety, M → FAQLQ, Y)

We developed a mediational model based on the associations, assessing whether attitudes towards FA could explain FAQL and whether these relations are mediated by FA anxiety. The results are shown in Table 3, and the mediational model is shown in Figure 3. The total effects (X → Y) column refers to the influence of attitudes towards FA (independent variable, X) on the FAQLQ subscales (dependent variable, Y). The indirect effects (X → M → Y) column refers to the effects of attitudes towards FA on the FAQLQ factors through FA anxiety (mediator variable, M). Finally, the direct effects assess the remaining effect of attitudes towards FA on the FAQLQ factors after including FA anxiety as a mediator. As can be seen, attitudes towards FA significantly predict all FAQL subscales. When FA anxiety is included as a mediator, the indirect effects of attitudes on FAQLQ remained significant. However, the direct effects were no longer significant.

**TABLE 3:** Mediational model coefficients: Attitudes (X) → food allergy (FA) anxiety (M) → food allergy quality of life (FAQL) (Y)

FAQL factors	Total effects (X → Y)	Indirect effects (X → M → Y)	Direct effects (remaining X → Y)
Emotional impact	0.37 [0.21, 0.53]**	0.30 [0.18, 0.43]**	0.07 [−0.03, 0.17]
FA Health	0.33 [0.19, 0.47]**	0.26 [0.16, 0.38]**	0.07 [−0.01, 0.15]
Risk	0.30 [0.16, 0.46]**	0.28 [0.17, 0.42]**	0.07 [−0.01, 0.15]
Social dietary limitations	0.34 [0.19, 0.50]**	0.29 [0.17, 0.43]**	0.07 [−0.01, 0.15]

*Note*: **p < 0.001.

## 6 DISCUSSION

People make positive and negative evaluations daily, based on the amount of cognitive, affective, and behavioral information they have about a specific object or topic.^8^ These judgments that can vary in valence and strength are known as attitudes.^8^ Researchers have assessed the significant impact of attitudes when focused on or associated with many different diseases and disorders, such as epilepsy,^13^ respiratory illness,^14^ and autism.^15^ However, the proper assessment and study of attitudes considering their psychological components are still incipient in FA research. Thus, the first step to further understanding attitudes toward FA is to develop a reliable and precise psychometrical tool for FA. Therefore, the present research aimed to develop the ATFAS.

### 6.1 Structure and item parameters

First, we assessed the ATFAS′ structure, and tested whether it would hold using robust statistical analysis. An EFA (Study 1) indicated the preference for a one‐dimensional structure, that presented strong results across groups of non‐allergic individuals and food‐allergic. We aimed to assess the structure across different groups to guarantee its applicability beyond those directly affected by the disorder. Knowing this would help to confidently use ATFAS in different groups indirectly affected by FA, such as parents and caregivers, and even those in the food business, such as restaurant managers. Moreover, we assessed the unidimensional structure through Confirmatory Factor Analysis (Study 2), using a large sample of food‐allergic individuals. The structure presented a good model fit, indicating that attitudes towards FA can be confidently assessed using a single factor. The measure also showed good reliability levels across all the samples and studies, using two well‐known methods (McDonald's omega), Cronbach's alpha.^23^

Furthermore, we evaluated discrimination, difficulty, and information levels for each item that constitutes the ATFAS (Study 1). Assessing these parameters helps to identify whether the measure items are properly helping to assess attitudes, which is central to validity. All items showed very high discrimination values,^25^ distinguishing individuals with various levels of attitudes towards FA. If an individual has negative attitudes toward the disorder and another has highly positive attitudes, the ATFAS′ items are sufficient to differentiate them based on their answers. These items also showed difficulty levels within the recommended threshold.^26^ If an item is too easy, most participants would highly endorse it. In contrast, if an item is too difficult, only those with high latent trait levels (i.e., in this case, with extremely positive attitudes towards FA) would endorse it. Therefore, items that are not too easy or too difficult are preferable to avoid skewed answers from participants. The ATFAS items can be used for individuals with differing levels of attitudes. Finally, all items presented a reasonable amount of information, individually and across all groups. A measure that presents higher information is also more reliable,^27^ corroborating our findings in the reliability analyses. Overall, our results support the unidimensional assessment of attitudes towards FA, with items that present parameters within the recommended threshold.

### 6.2 Associations between ATFAS and food allergy anxiety and FAQLQ

Using a subsample in Study 2, we assessed the associations between ATFAS, FA anxiety, and FAQL. Attitudes presented significant negative associations to FA anxiety and significant positive to all FAQLQ subscales. In effect, individuals with negative attitudes towards the disease are also more likely to have a higher level of FA anxiety, whereas more positive attitudes are linked to a higher quality of life. Previous research has also shown a link between negative attitudes and anxiety,^13^, ^32^ and between positive attitudes and improved quality of life across different settings.^33^, ^34^ Our research's positive associations between FAQL and attitudes are novel, which may have implications for clinical practice and/or intervention programs. For instance, the social stigma surrounding FA enhances the difficulties faced by parents of children with FA when dealing with the disorder.^35^ Therefore, researchers and clinicians might help develop interventions that aim to develop positive attitudes among these parents, which could help reduce the burden of this stigmatization and improve the parents' and their children's quality of life. This focus on a more positive attitude towards the disease might also be beneficial in school settings, as it is known that the ‘FA label’ increases the chances of being a victim of bullying.^36^ For instance, through interventions that focus on understanding children's views towards the disease and how they think these can change towards a more positive, inclusive, and friendly environment.

Moreover, based on the significant associations between the variables, we also assessed whether the relations between attitudes towards FA and FAQL could happen through FA anxiety. More specifically, more positive attitudes towards FA influence FAQL. However, these direct influences turn non‐significant after including FA anxiety within the model. Such findings highlight the indirect critical role of FA anxiety when developing positive attitudes to enhance FAQLQ. That is, positive attitudes toward FA help reduce FA anxiety, which in turn helps to improve FAQL. Our findings align with the attitudes literature and its association with psychological indicators such as anxiety and quality of life. For instance, positive attitudes toward epilepsy significantly reduce anxiety and improve quality of life.^37^ Therefore, when considering the impact positive attitudes toward FA might have on developing a greater FAQL, it is essential to consider that these effects might happen by reducing the FA anxiety levels.

### 6.3 Limitations and final considerations

Despite our relevant findings, it is essential to highlight some potential limitations in our studies. For instance, we did not consider whether participants from the non‐diagnosed group had any close friends or relatives with FA. This familiarity with the disease could influence their attitudes towards FA, either positively or negatively. Another potential limitation is that most participants were diagnosed by a GP or family doctor, whereas a specialist (allergologist) would be preferable. We also did not ask about their procedure for diagnosis. Furthermore, we used a subsample for our convergent analyses rather than the entire sample—although the subsample size provides enough power for the analyses performed. Finally, using cross‐sectional data might raise some caution in interpreting our findings.

Our research attempted to develop the first measure of attitudes towards FA, which applies both to patients and the general population. A reliable and precise psychometrical tool is necessary to allow a more in‐depth understanding of how attitudes can impact the lives of those directly and indirectly affected by FA. We are confident that the ATFAS is a novel and necessary tool that can help to broaden our perspective and widen our knowledge of the FA. Also, as previously stated, the use of an attitudinal scale might contribute to clinical practice and/or intervention programs. For instance, understanding how to develop more positive attitudes towards the disease could help reduce the burden many individuals face daily and improve their quality of life, as seen in other contexts.^33^, ^34^

## AUTHOR CONTRIBUTIONS

Gabriel Coelho and Renan Monteiro wrote the first draft of the manuscript, which was then reviewed, amended, and approved by all co‐authors.

## CONFLICT OF INTEREST

The authors have no conflict of interest to declare.

## INFORMED CONSENT

Informed consent was obtained from all participants.

## CONSENT FOR PUBLICATION

All authors provided input into the manuscript, reviewed the final draft and provided consent for publication.

## Attitudes Towards Food Allergy Scale

Please, read the following statement and indicate your attitudes towards FA, using the answering scale below, from −3 to +3. Answers placed closer to one end of the scale should be more representative of that end, while answers placed in the middle are neutral.

I see food allergy as…

**Table:**
